# Women´s expectations and evaluation of a maternal educational program

**Published:** 2013-09-30

**Authors:** Juan Miguel Martínez, Miguel Delgado

**Affiliations:** 1 Andalusian Health Service. University of Jaen.The Biomedical Research Centre Network for Epidemiology and Public Health (CIBERESP). Spain; 2 University of Jaen, Department of Health Sciences, Department of Health Sciences, University of Jaén. CIBERESP.Spain

**Keywords:** Health education, patient satisfaction, required request, pregnancy, prenatal care

## Abstract

**Objectives:**

To identify the expectations of women requesting the maternal education program (ME) and to determine their evaluation of it.

**Methods:**

a multi-centric observational study was conducted in four hospitals in Spain in 2011 with primiparous women. Socio-demographic and obstetrical variables, among others, were collected through interviews and reviews of medical records. The analysis estimated crude and adjusted odds ratios using logistic regression with a confidence interval of 95%.

**Results:**

Newborn care was most requested type of content desired by women (80.33%). Eleven and one quarter percent (11.25%) of the women evaluated ME as being of little or no usefulness or benefit. Women appreciated the follow-up care given during pregnancy and childbirth but ME was not noted as influencing the measurement of these processes (*p*> 0.05).

**Conclusions:**

Newborn care was the type of subject mainly demanded by the women in the ME program. Women evaluated ME as being a useful program.

## Abstract (es)

**Objetivos:**

Identificar las demandas que solicita la mujer del programa de educación maternal (EM) y determinar la valoración que hacen de este.

**Métodos:**

Estudio multicéntrico observacional llevado a cabo en cuatro hospitales de España en 2011 con mujeres primíparas. Mediante entrevista y revisión de la historia clínica se recogieron diferentes variables sociodemográficas, obstétricas, entre otras. En el análisis se estimaron odds ratios crudas y ajustadas mediante regresión logística con un intervalo de confianza del 95%.

**Resultados:**

El cuidado del recién nacido fue el tema más solicitado por la mujer (80.33%). El 11.25% de las mujeres valoraron como poco o nada útil y beneficiosa la EM. Las mujeres valoraron positivamente el control de su embarazo y la atención recibida en su parto pero no se detectó influencia de la EM en la valoración de estos procesos (*p >*0.05*).*

**Conclusiones:**

El cuidado del recién nacido es el tema que las mujeres demandan mayoritariamente del programa de EM. Las mujeres valoran como útil la realización de la EM.

## Introduction

Maternal Education (ME) is a health education program provided during pregnancy, labor and childbirth that should positively impact the health of mothers and children; therefore, it encompasses a range of educational and support measures that help couples and parents to understand their own social, emotional, psychological and physical needs during pregnancy, labor and parenthood^1^. In Spain this program is part of the service portfolio of the national health system offering universal and free access, i.e. it is provided at all health centers to all users.

It is mainly carried out in group setting sessions in the third trimester of pregnancy and includes lifestyle norms, theory of pregnancy, physical and psychological preparation for delivery and newborn care, as well as other elements (chat groups and physical exercises). At a minimum, at least three sessions are mandatory and begin in the third trimester^2^.

Given the current status of ME, several studies have recommended redesign and current ME program evaluations and advise that new strategies and pedagogical approaches should be considered^3^ ^-^ ^5^. Moreover, midwives working in primary care and therefore mainly responsible for providing or developing the ME program are aware that they cannot avoid the social changes taking place in such a complex process as the field of maternal and family health. Hence, they must make adjustments to the content and methodology used to adapt ME programs to the new societal demands, and to program users^6^. In fact, in the study done by Gallardo and Sánchez^7^ the majority of midwives (91%) reported that there was a change in expectations by the population in terms of ME and 88% of these health professionals stated that they had made ​​adjustments to the ME program.

No recent studies were found that analyzed the expectations of women in the ME program. There are changes in the perinatal care context made by health administration and this administration also proposes an increasingly central role for citizens that will serve as the basis for the development of new strategies^8^. An additional consideration is that very few women are attending the current ME program^9^ ^-^ ^11^. For all these reasons, the need arose to identify the expectations of women for the ME program, their evaluation of it, as well as to know the effect of ME on user satisfaction with follow-up on the pregnancy and care received at delivery.

## Materials and Methods

A multi-centric observational study was conducted between January 2011 and January 2012 at the healthcare centers in the province of Jaén (University Hospital Complex of Jaén and the San Juan de la Cruz de Úbeda Hospital), and at the Poniente in El Ejido (Almería) Hospital and at the University Hospital Virgen de las Nieves of Granada, all of which are located in southern Spain. The reference population was women who gave birth at one of these centers and that met the following inclusion criteria: primiparous, single pregnancy and over 18 years of age. The study was approved by the Ethics Committees of the respective centers and informed consent was required. A language barrier was established as an exclusion criterion.

The estimation of the sample size was based on the following assumptions. The percentage attending ME was one-third of the total^9^ ^-^ ^11^. The general intent of the study was to reduce the number of cesareans, and they fell from 20% to 10% among those receiving ME^12^ ^,^ ^13^. A a power of 80% and an alpha error of 5% were established, and 507 women were required for the study. The women were selected consecutively.

Information was collected after childbirth on socio-demographic data (age, sex, marital status, nationality, income, highest educational level achieved, work done during pregnancy, type of contract, sector in which work was done, race and nationality), as well as variables describing the presence of any pathology during pregnancy, pregnancy intent, level of health care provided during pregnancy, adequate prenatal care received (≥4 visits for pregnancy follow-up care and the first visit made before the 12th week of pregnancy), abortion history, personal history of disease, prior intent before pregnancy to participate in the ME program, expectations of the women concerning the ME program, user satisfaction with the management of the pregnancy and with the care received during the delivery, and an assessment made by ME participants. For the last three variables a 5-point Likert scale (0-4) was used. The data were collected from an interview of the woman and were validated by the maternal clinical history and medical records. The questionnaire consisted of 140 items (130 closed and 10 open questions) and was applied by 24 previously trained interviewers.

In the data analysis, the odds ratio (OR) was estimated for dichotomous variables and the confidence intervals (CI) was set at 95%. Logistic regression was applied in the multivariate analysis retaining as confounding variables those that altered the main exposure coefficient by more than 10%; as potential *a priori *confounders, the socio-demographic characteristics of the women and the presence of pathology during pregnancy was considered. When the outcome variable was continuous (e.g., degree of user satisfaction with the management of pregnancy) a comparison of means was used, and the multivariate analysis used an analysis of covariance that adjusted for the same variables.

## Results

The study involved 520 women, of which 357 (68.65%) had gone to the ME program. The characteristics of the women who participated in the study were as follows: they had a mean age of 29.91 + 5.30 years, 97.88% were white and 64.7% were married; also, 89.62% were Spanish nationals. Of these women, 31.73% had studied at the university level and 46.94% had a monthly income between 1,000-1,999 Euros. 87.50% of these women were healthy before pregnancy and, in 90% of the cases, the pregnancy was sought. 77.50% of women were having their pregnancy monitored through primary care and 91.80% had good prenatal care.

Table 1 documents the association between those having had ME and expectations for issues to be addressed according to participant suggestions. In this regard, of the 427 women that responded, 80.33% of all women wanted the issue of newborn care addressed. 78.57% of women who did not think that newborn care was relevant had received maternal prenatal ​​care, compared to 71.4% who felt it was important (adjusted OR= 0.69, 95% CI= 0.38-1.27). Another fundamental content issue that women thought necessary in the ME program was breastfeeding in which 74.71% of the women expressed support. 

**Table 1:**
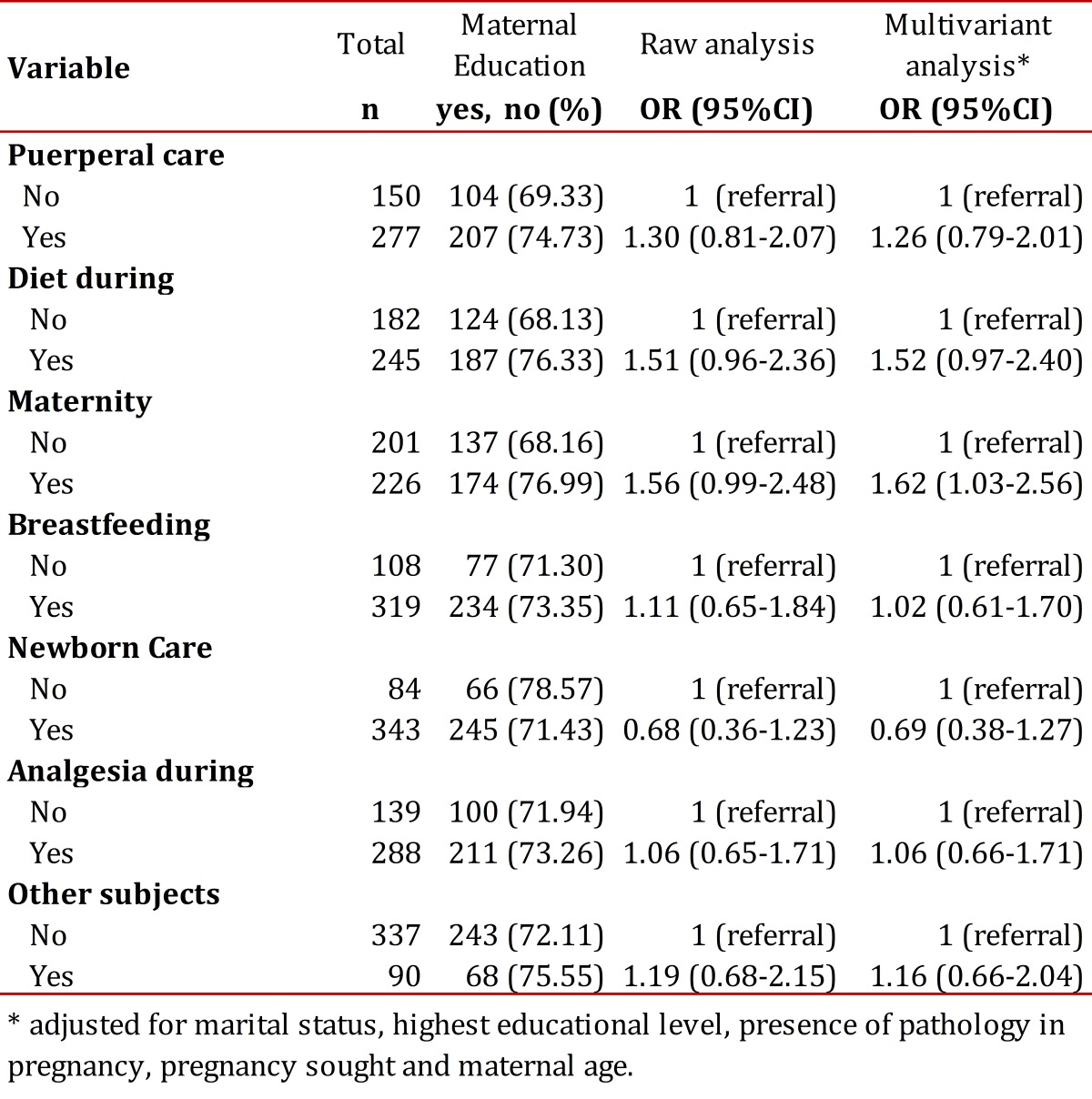
Association between attendance for maternal education and subject matter expected by women for this program

Of the women who reported breastfeeding as a necessary topic for discussion in ME, 73.35% participated in the ME program, while 71.30% of the women who believed that training on this issue was not needed attended ME sessions (OR= 1.11, 95% CI= 0.65-1.84); after adjustment for marital status, educational level, presence of pathology in pregnancy, sought pregnancy and maternal age, there were no significant differences found (OR= 1.02, 95% CI= 0.61-1.70). 21.08% of the women highlighted as topics to be addressed within the ME program a variety of subjects, including straining, physical exercise, etc. As for the number of issues to be addressed in ME classes there was no significant difference between the group of women who had received ME and those who had not gone through the program (*p*= 0.092).

Table 2 shows that 88.75% of the women acknowledged the usefulness and benefit of attending ME sessions, and there is a positive association between the higher the grade given to its usefulness and participant follow through with maternal education (*p* <0.001) .

**Table 2.**
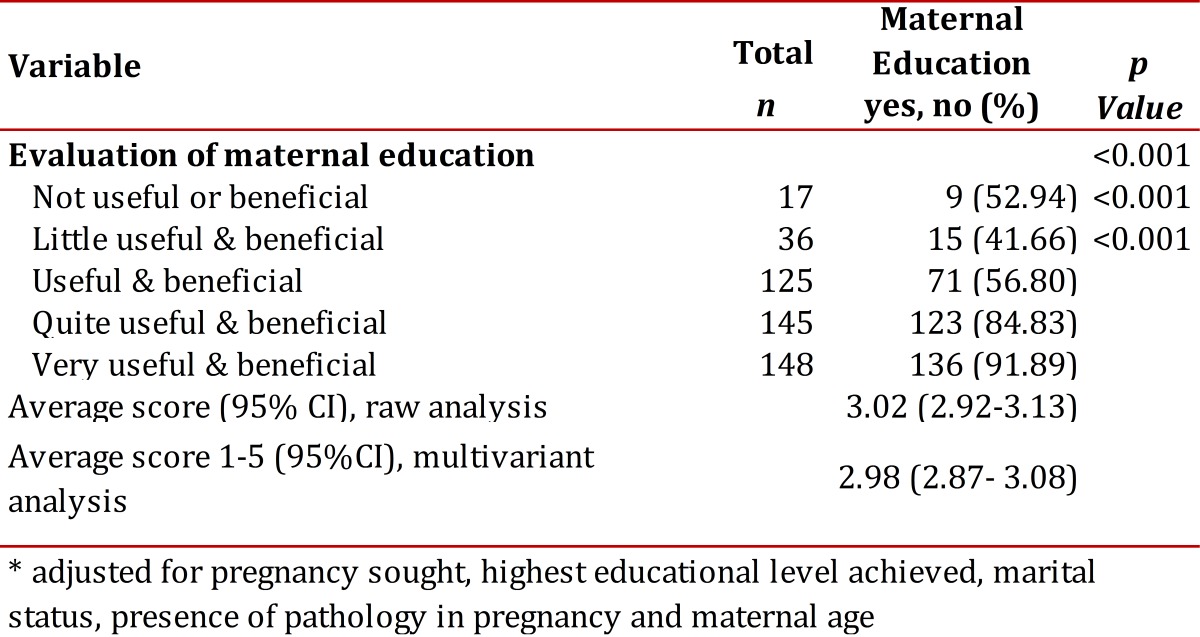
Association between the usefulness that women attribute to maternal education and program participation

The degree of satisfaction of women with pregnancy follow-up care and with care received during delivery is quite high, with scores around 3 to a maximum of 4 as can be seen in Table 3. No relationship was observed between participation in the ME program and the degree of satisfaction with care given during pregnancy (*p* = 0.488), or the degree of satisfaction with the care provided at delivery (*p*= 0.777) (Table 3). 63.71% of the women who participated in the ME program reported to be very or fairly satisfied compared to 10.53% who indicated being little satisfied or dissatisfied with ME.

**Table 3:**
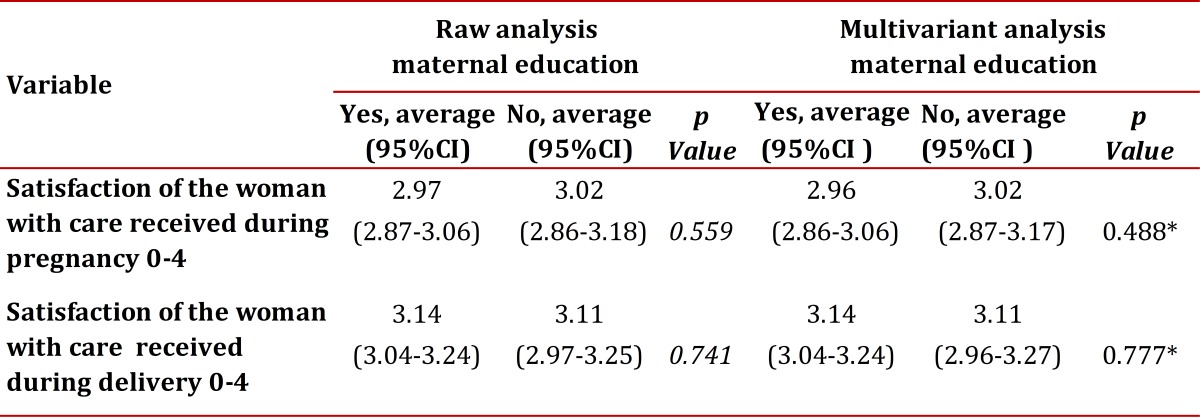
Association between the woman´s degree of satisfaction with follow-up care and with care received during the birthing process and participation in maternal education

## Discussion

In general, most women asked for training on newborn care as a subject to be addressed in the ME program. This finding is consistent with other studies^1^. The topic with the next greatest demand for information concerned breastfeeding, followed by the issue of pain relief during labor, as has been found by other authors^14^. It was not found that the demand for certain subjects, such as diet, postpartum care, etc. or the number of participants demanding it was influenced by ME attendance.

Health professionals involved with the process of pregnancy, labor and birth can leverage the opportunity provided by ME to sensitize women to the importance of issues such as the postpartum period because, despite the importance of this period and the level of care generally required, it seems that once delivery has occurred and the process finished, lowered levels of alertness and care needs tend to minimize the importance of this period for users^15^.

Along this line, most postpartum women could benefit from more training acquired through ME for useful postpartum practices and those that may have adverse effects on the health of the mother and the newborn^16^. Among the benefits that support postpartum approaches being incorporated into the ME program is the prevention of a health problem of great magnitude with important consequences: postpartum depression^17^.

A relationship was also not detected between attending ME classes and the degree of satisfaction of women with the follow-up pregnancy care received, or with care received during delivery. Here it worthy to note that women who attended the ME gave a lower evaluation, not statistically significant, to follow-up care received during pregnancy than did those that did not attend. Also, there was no significant relationship found with care received during delivery. Therefore we cannot conclude that ME influenced the degree of satisfaction with the management of pregnancy and delivery care, contrary to what is mostly found in the scientific literature^18^ ^-^ ^22^, although another Spanish study^5^ found results similar to ours.

In most cases, women positively evaluated ME, both considering it useful and beneficial. This evaluation increases with program attendance and is in agreement with other studies^1^ ^,^ ^23^. The evaluation of the ME program is of vital importance for involving women with health related issues both in the process of pregnancy, labor and childbirth and in other general aspects of health throughout their lifetime^24^.

If there was a selection bias associated with non-response, it will have had minimal influence on the validity of the results since there is no a priori reason to think that the women who responded substantially differed from non-responders. Nor is it believed very likely that a misclassification bias was introduced because different questions in the questionnaire were completed by the collaborating personnel who previously had been trained not to note different interpretations of the question on the part of the women.

In conclusion, women chiefly expected the subject of newborn care to be a topic to be dealt with at sessions of the ME program. The ME program has been found to be useful for women, whether they make use of it, or not. The ME program does not seem to influence the evaluation of prenatal care processes and birthing, they are independent.
